# Environmental Investigation of a Campylobacteriosis Outbreak Among Wedding Attendees — York County, Pennsylvania, 2025

**DOI:** 10.15585/mmwr.mm7537a2

**Published:** 2026-09-24

**Authors:** Rugiatu Z. Kamara, Shanna Miko, Amy M. Kahler, Alexis V. Roundtree, Haley J. McKeel, Yang Gao, Charlotte Lane, Kristina Zwolenik, Kelly E. Kline, Aaron Smee, Olivia Fitzmaurice-Shean, Bevin S. Durant Fidler, Nicholas Cesari, Shannon McGinnis, Mia Catharine Mattioli

**Affiliations:** ^1^Division of Foodborne, Waterborne, and Environmental Diseases, National Center for Emerging and Zoonotic Infectious Diseases, CDC; ^2^Epidemic Intelligence Service, CDC; ^3^Chenega Enterprise Systems & Solutions, LLC, Chesapeake, Virginia;^ 4^Pennsylvania Department of Health, Bureau of Epidemiology; ^5^Pennsylvania Department of Agriculture, Bureau of Food Safety & Laboratory Services.

**Summary**

**What is already known about this topic?**

Unregulated private springs are a known risk factor for gastrointestinal illness, including campylobacteriosis.

**What is added by this report?**

A *Campylobacter jejuni* outbreak among wedding attendees in Pennsylvania was linked to consumption of untreated water from an on-site spring; environmental sampling detected *Campylobacter* DNA in the spring and a tap it supplied. The broken springhouse roof permitted entry of debris and animal feces, an ultraviolet water treatment light was nonfunctional, and evidence of animal activity was observed in and around the spring.

**What are the implications for public health practice?**

Routine inspection and maintenance of untreated private springs and clear signage indicating whether water is potable might reduce the risk for waterborne outbreaks. Pairing environmental testing with epidemiology helps confirm exposure routes and can guide mitigation efforts.

In early June 2025, approximately 80 persons attended a wedding at a venue in York County, Pennsylvania. Within 3 days, multiple attendees reported experiencing gastrointestinal illness. On June 23, the Pennsylvania Department of Health (PDOH) and the Pennsylvania Department of Agriculture (PDA) contacted CDC to request assistance after PDOH became aware of a laboratory-confirmed *Campylobacter jejuni* infection and reports of approximately 20 additional symptomatic attendees, with epidemiologic information implicating exposure to untreated spring water at the venue as a common exposure source. The focus of this report is on environmental inspection, testing, and identification of pathogens. This activity was reviewed by CDC, deemed not research, and conducted consistent with applicable federal law and CDC policy.*

## Investigation and Outcomes

### Survey of Event Attendees

On June 30, PDOH sent a link to an online questionnaire to all wedding attendees to identify additional cases and characterize exposures. Because some attendees shared email addresses, the exact number of attendees who received the questionnaire could not be determined; however, 46 responses were received. Among these, 15 (33%) persons reported gastrointestinal illness 1–10 days after the event; however, no specific food item associated with illness was identified.

### Use of On-Site Spring Water During the Event and Inspection of the Spring

The venue manager reported that the site normally relied on vendors to supply potable water and ice. However, after the vendor’s bottled water supply was exhausted during the event, employees of the vendor began drawing water from a kitchen tap connected to an on-site spring for food preparation and beverage service; no signage identified the water as nonpotable.

An onsite inspection by PDOH and PDA officials identified substantial deficiencies at the spring, including 1) a broken springhouse roof allowing entry of debris and animal feces (Figure), 2) a nonfunctional ultraviolet treatment unit (inoperable for ≥2 months), 3) evidence of animal activity, and 4) proximity of the spring to a chicken coop and an area occupied by horses.

**FIGURE:**
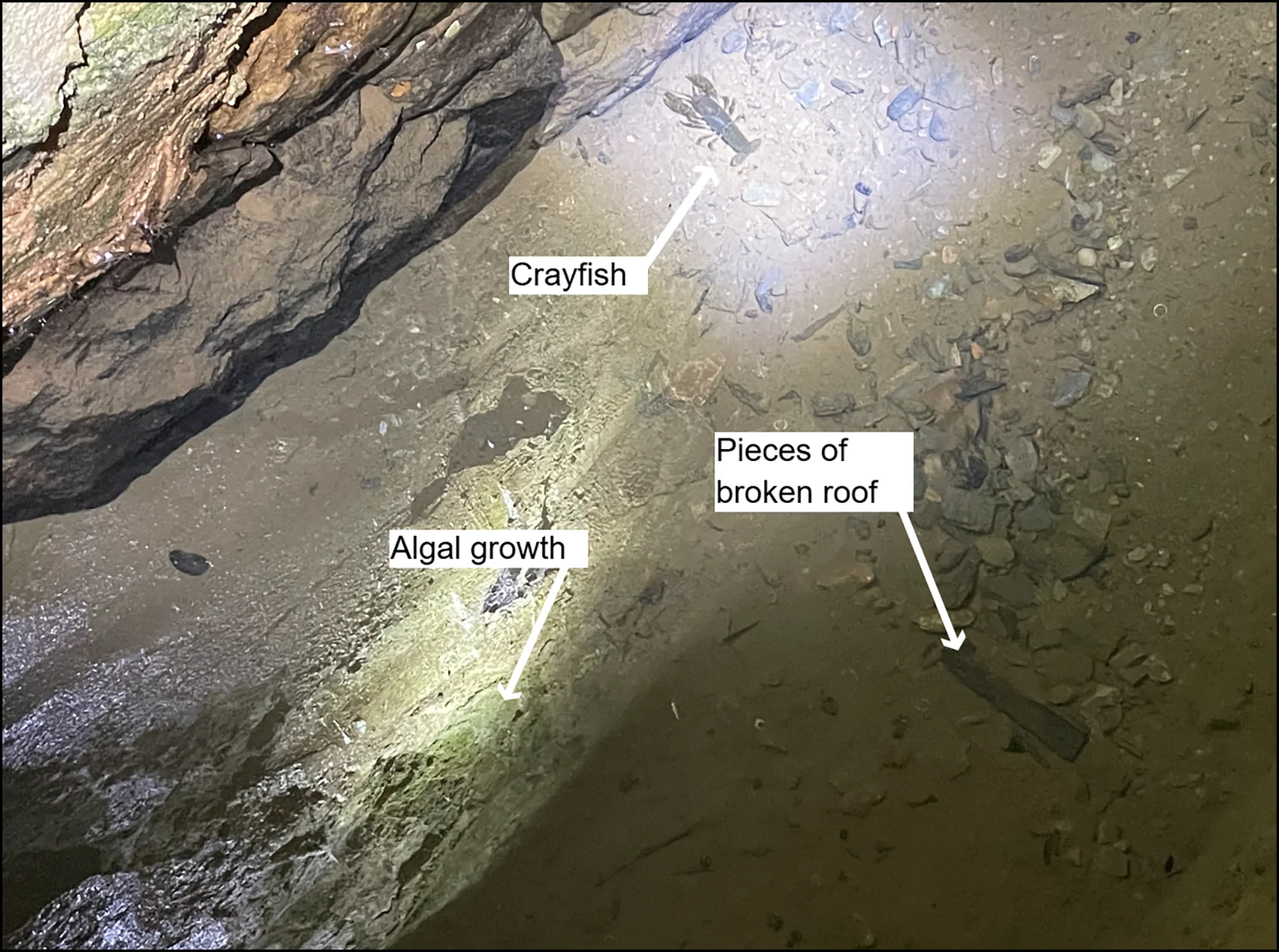
Bottom of the spring that provided water to a wedding venue and was associated with an outbreak of *Campylobacter jejuni* gastroenteritis, showing algal growth, pieces of the broken roof, and evidence of animal activity — York County, Pennsylvania, 2025 Photo/Pennsylvania Department of Health, 2025

### Testing of Water Samples

On July 9, PDOH and PDA officials collected paired large-volume (100-L) ultrafiltered and (500-mL) grab samples^†^ from the spring and the kitchen tap (*1*); point-of-use ultraviolet filters^§^ had been installed in the kitchen tap after the wedding but before sample collection. CDC’s Environmental Microbiology and Engineering Laboratory analyzed samples for *Campylobacter* spp. (*2*), culturable fecal indicator bacteria (total coliforms and *Escherichia coli*), and microbial source tracking^¶^ molecular markers targeting fecal contamination from birds and ruminants (*3*,*4*), potential reservoirs of *Campylobacter* organisms present on the property.

Culturable *Campylobacter* organisms were not recovered from any sample, but *Campylobacter* DNA was detected in both spring and tap water. Fecal indicator bacteria were markedly elevated in spring water (total coliforms >2,419.6 most probable number [MPN]/100 mL; *E. coli* 547.5 MPN/100 mL)** but undetectable at the tap, suggesting that point-of-use treatment reduced but did not eliminate detectable *Campylobacter* DNA. Source tracking markers for birds and ruminants were not detected at either site, leaving the source or sources of fecal contamination undetermined.

## Preliminary Conclusions and Actions

Epidemiologic linkage of illness to spring-water consumption, environmental deficiencies at the spring, and detection of *Campylobacter* DNA and fecal indicator bacteria in the spring water support untreated spring water as the most likely outbreak source. Absence of culturable pathogens does not rule out prior contamination, because *Campylobacter* organisms can enter a viable but nonculturable state (*5*), and sampling occurred after symptom onset. After the investigation, the venue installed two point-of-use water filters, repaired the springhouse roof, and posted signage identifying water from spring-fed taps as nonpotable. State officials recommended that the venue improve communication with external vendors to prevent future use of the nonpotable water source for food preparation and drinking.

Private springs are largely unregulated in the United States, and, in the absence of visible contamination, users might assume such water is safe. These findings suggest that timely environmental testing paired with epidemiologic investigation could improve source attribution and guide the development and implementation of remediation efforts at venues serving the public. Routine inspection and maintenance of untreated private water sources might reduce the risk for waterborne outbreaks.

## Footnotes

* 45 C.F.R. part 46, 21 C.F.R. part 56; 42 U.S.C. Sect. 241(d); 5 U.S.C. Sect. 552a; 44 U.S.C. Sect. 3501 et seq.

† A single discrete sample collected at a specific location to provide a measurement of contamination at a specific point in time.

§ Ultraviolet treatment systems use ultraviolet light to disinfect water or reduce the number of harmful microorganisms in water.

¶ Microbial source tracking uses tests that identify fecal bacteria linked to specific hosts to help determine where contamination in environmental water comes from.

** Measured using Environmental Protection Agency standard methods, IDEXX Colilert-18, on 100-mL grab samples.
